# Desenvolvimento de uma medida de letramento funcional em saúde: aplicação da Teoria da Resposta ao Item

**DOI:** 10.1590/0102-311XPT060225

**Published:** 2026-02-13

**Authors:** Patricia Romualdo de Jesus, Tatiane da Silva Dal Pizzol, Tatiana da Silva Sempé, Sotero Serrate Mengue, Stela Maris de Jezus Castro

**Affiliations:** 1 Programa de Pós-graduação em Epidemiologia, Universidade Federal do Rio Grande do Sul, Porto Alegre, Brasil.; 2 Programa de Pós-graduação em Assistência Farmacêutica, Universidade Federal do Rio Grande do Sul, Porto Alegre, Brasil.

**Keywords:** Letramento em Saúde, Psicometria, Análise de Classes Latentes, Health Literacy, Psychometrics, Latent Class Analysis, Alfabetización en Salud, Psicometría, Análisis de Clases Latentes

## Abstract

Este estudo teve como objetivo desenvolver uma medida de letramento funcional em saúde (LFS), utilizando a Teoria da Resposta ao Item (TRI); coletar evidências de validade da medida criada (validade convergente); e propor pontos de corte para o traço latente de LFS. Foi realizado um estudo metodológico, utilizando dados de um estudo transversal de base populacional. A coleta de dados ocorreu entre outubro de 2023 e março de 2024, em domicílios de cinco municípios brasileiros. Para a criação do Teste de Letramento Funcional em Saúde (TLFS), foi utilizada uma versão reduzida e adaptada do Teste de Letramento em Saúde. Para o desenvolvimento da medida, foi usado o Modelo de Resposta Gradual de Samejima, com aplicação da TRI. A validade convergente foi avaliada por meio da comparação das medidas de LFS com as variáveis idade, escolaridade e classe econômica. O modelo de classes latentes foi empregado para categorizar os escores gerados pela TRI. A amostra foi composta por 1.181 participantes, metade apresentando idade entre 30 e 59 anos. Todos os itens do TLFS apresentaram boa capacidade de discriminação. A análise de validade convergente demonstrou uma diminuição significativa nas médias do traço latente de LFS entre os participantes com idade mais avançada, baixa escolaridade e de classes socioeconômicas mais baixas. O modelo de três classes latentes apresentou o melhor ajuste, sendo definidos três níveis de LFS: inadequado, limitado e adequado. O TLFS foi disponibilizado em um aplicativo, sendo acessível para uso em pesquisas e na prática clínica. Os resultados indicaram um bom desempenho psicométrico na avaliação do nível de LFS, fornecendo as primeiras evidências de validade do instrumento TLFS.

## Abstract (en)

This study aimed to develop a measure of functional health literacy (FHL) using Item Response Theory (IRT) to collect validity evidence of the created measure (convergent validity) and to propose cut-off points for the latent FHL trait. A methodological study was carried out with data from a population-based cross-sectional study. Data were collected from October 2023 to March 2024 in households in five Brazilian municipalities. A reduced and adapted version of the health literacy test was used to create the Functional Health Literacy test (FHLT) with the Samejima Gradual Response Model and IRT. Convergent validity was assessed by comparing functional health literacy measures with age, schooling, and economic class. The latent class model was used to categorize the scores generated by IRT. The sample consisted of 1,181 participants, half of whom were aged from 30 to 59 years. All FHLT items showed good discrimination. Convergent validity analysis showed a significant decrease in the means of the latent functional health literacy trait in participants with older age, low education, and lower socioeconomic classes. The model of three latent classes showed the best fit, defining three functional health literacy levels: inadequate, limited, and adequate. The FHLT was made available on an app for research and clinical practice. The results indicated the good psychometric performance of the FHLT in assessing functional health literacy levels, providing the first evidence of its validity.

## Abstract (es)

El objetivo de este estudio fue desarrollar una medida de alfabetización funcional en salud utilizando la Teoría de Respuesta al Ítem (TRI), recopilar pruebas de la validez de la medida creada (validez convergente) y proponer puntos de corte para el rasgo latente de alfabetización funcional en salud. Se realizó un estudio metodológico utilizando datos de un estudio transversal de base poblacional. La recopilación de datos se llevó a cabo entre octubre de 2023 y marzo de 2024 en hogares de cinco municipios brasileños. Para la creación de la Prueba de Alfabetización Funcional en Salud (PAFS), se utilizó una versión reducida y adaptada de la Prueba de Alfabetización en Salud. Para el desarrollo de la medida, se utilizó el Modelo de Respuesta Gradual de Samejima, con la aplicación de la TRI. La validez convergente se evaluó comparando las medidas de alfabetización funcional en salud con las variables: edad, nivel educativo y clase económica. Se utilizó el modelo de clases latentes para categorizar las puntuaciones generadas por el TRI. La muestra se compuso de 1.181 participantes, la mitad de los cuales tenían entre 30 y 59 años. Todos los ítems de la PAFS mostraron una buena capacidad de discriminación. El análisis de validez convergente demostró una disminución significativa en las medias del rasgo latente de alfabetización funcional en salud entre los participantes de mayor edad, con bajo nivel educativo y de clases socioeconómicas más bajas. El modelo de tres clases latentes presentó el mejor ajuste, definiéndose tres niveles de alfabetización funcional en salud: inadecuado, limitado y adecuado. La PAFS se puso a disposición en una aplicación, haciéndola accesible para su uso en investigaciones y en la práctica clínica. Los resultados indicaron un buen rendimiento psicométrico en la evaluación del nivel de alfabetización funcional en salud, proporcionando las primeras pruebas de la validez del instrumento PAFS.

## Introdução

O letramento funcional em saúde (LFS) abrange as habilidades básicas de leitura e escrita que um indivíduo deve possuir para agir de maneira eficaz em situações cotidianas relacionadas à saúde ^1^. No entanto, essas habilidades não são os únicos fatores que determinam o LFS, uma vez que essa competência também envolve a capacidade de buscar, interpretar e utilizar informações dentro de um sistema de saúde ^2^. 

Diversos instrumentos são utilizados para avaliar diferentes dimensões do letramento em saúde. No Brasil, foram identificados mais de 50 estudos medindo o letramento em saúde, sendo que a maioria focou no LFS a partir da avaliação de habilidades como compreensão de texto e numeramento ^3^. Um exemplo é o Teste de Letramento em Saúde (TLS) ^4^, adaptado transculturalmente e validado para o português do Brasil a partir do *Test of Functional Health Literacy in Adults* (TOFHLA), um instrumento americano amplamente utilizado para a avaliação do LFS. Apesar dessa diversidade, muitos instrumentos exigem um tempo prolongado para sua aplicação, tornando-os pouco práticos em contextos que demandam agilidade na avaliação. Nesse contexto, a Teoria da Resposta ao Item (TRI) tem sido uma das metodologias empregadas no desenvolvimento ou adaptação de instrumentos de medida. No Brasil, essa metodologia tem sido utilizada para avaliar as propriedades psicométricas de versões adaptadas para o português de instrumentos que medem o letramento em saúde ^5^ ^,^ ^6^ ^,^ ^7^. Até o momento, não foram identificados estudos brasileiros que tenham utilizado a TRI para avaliar instrumentos de mensuração do LFS. Já em outros países, essa abordagem tem sido amplamente utilizada, tanto em análises psicométricas de novas medidas ^8^ ^,^ ^9^ quanto em processos de adaptação ^10^ e redução ^11^ de instrumentos que avaliam o letramento em saúde ou LFS. Assim, o desenvolvimento de instrumentos mais curtos, fundamentados em metodologias robustas como a TRI, pode oferecer uma avaliação mais precisa e aplicável do LFS.

O objetivo principal deste trabalho foi desenvolver uma medida do traço latente de LFS utilizando a TRI. Os objetivos secundários foram coletar evidências de validade da medida criada (validade convergente) e propor pontos de corte para o traço latente de LFS a partir da técnica de Análise de Classes Latentes (LCA, acrônimo em inglês), permitindo classificar os indivíduos de acordo com os seus níveis de LFS.

## Métodos

### Delineamento, local e população do estudo

Este é um estudo metodológico com dados provenientes de um estudo transversal de base populacional. A coleta de dados ocorreu entre outubro de 2023 e março de 2024, em domicílios de cinco municípios selecionados por conveniência: Campo Grande e Dourados (Mato Grosso do Sul), Pelotas, Porto Alegre e Santa Maria (Rio Grande do Sul). Foram incluídos brasileiros com 18 anos ou mais, que sabiam ler e escrever.

Os dados foram obtidos por meio de uma entrevista estruturada, utilizando o aplicativo REDCap (https://redcapbrasil.com.br/) ^12^ ^,^ ^13^. Foram coletadas informações sobre sexo (feminino e masculino), idade (categorizada em 18-29 anos, 30-59 anos e mais de 60 anos), escolaridade (categorizada em 0-8 anos, 9-11 anos e mais de 12 anos), classificação econômica ^14^ (categorizada em estrato socioeconômico A e B, C e D e E). Foi realizada uma amostragem por conglomerados em três estágios: sorteio de setores censitários, quadras e domicílios. Dessa forma, as cinco cidades foram tratadas como um único domínio de estudo, permitindo a obtenção de estimativas para o conjunto de municípios, considerando sexo, faixa etária e escolaridade.

Os dados sociodemográficos foram apresentados como frequência relativa ponderada. As análises descritivas foram conduzidas no software RStudio 2024.12.0 (https://rstudio.com/), utilizando o pacote *survey* 4.4-2. 

### Teste de Letramento Funcional em Saúde

Para a criação do Teste de Letramento Funcional em Saúde (TLFS) usamos uma versão reduzida e adaptada do TLS ^4^, com autorização prévia dos autores. O TLS avalia as habilidades de leitura e numeramento relacionadas a informações de saúde, com um tempo médio de aplicação de 25 minutos. A seção de numeramento inclui 10 cartões que simulam instruções que um indivíduo pode receber em um estabelecimento de saúde, seguidos por 17 perguntas relacionadas às informações apresentadas. A seção textual consiste em três textos que utilizam o método *cloze* modificado, no qual o entrevistado deve preencher lacunas selecionando a alternativa correta. Essa seção é cronometrada, com tempo limite de 12 minutos. Os textos abordam temas como a preparação para um exame de raio-x, os direitos do cidadão no Sistema Único de Saúde (SUS) e um termo de consentimento informado, apresentando 16, 20 e 14 lacunas, respectivamente. 

A redução do instrumento envolveu uma análise de conteúdo das questões, realizada pela equipe de pesquisa do projeto, composta por pesquisadores com experiência na área de letramento em saúde e com o uso do instrumento TLS. A partir dessa análise, foi possível identificar itens com redundância de conteúdo e foram priorizados itens com maior clareza semântica e que contemplavam ambas as habilidades: numeramento e compreensão de leitura. Itens que apresentavam alta semelhança de conteúdo foram excluídos. Foram realizados pré-testes, com *feedback* dos participantes, para avaliar a compreensão das questões e garantir a objetividade e precisão do instrumento na avaliação do conteúdo proposto.

O novo instrumento de medida, denominado TLFS, foi composto por três cartões contendo um total de seis perguntas e um texto com orientações para um exame de raio-x (Material Suplementar − Figura S1; https://cadernos.ensp.fiocruz.br/static//arquivo/suppl-e00060225_9313.pdf). Esse texto foi dividido em três partes: preparação para o exame, orientações para o dia anterior e instruções para o dia do exame, totalizando 16 lacunas para preenchimento.

O método de correção das questões relacionadas aos cartões seguiu o pressuposto de independência local das questões. As questões 1 (“Se você toma seu primeiro comprimido às 6:00 da manhã, quando deveria tomar o próximo?”) e 2 (“E o próximo depois desse?”) apresentavam dependência local. Diante disso, a questão 2 foi considerada correta se o raciocínio de adição estivesse correto conforme a resposta da questão 1. As demais questões dos cartões foram corrigidas de acordo com o gabarito do TLS.

A correção do texto foi adaptada de acordo com sua divisão em três partes: a primeira com 5 lacunas (T1), a segunda com 6 lacunas (T2) e a terceira com 5 lacunas (T3). Cada parte do texto foi considerada como um único item e a pontuação foi definida da seguinte forma:

a) Para a parte com 5 lacunas: pontuação 1 = errou todas; pontuação 2 = acertou entre 1 e 3 lacunas; 

pontuação 3 = acertou entre 4 e 5 lacunas.

b) Para a parte com 6 lacunas: pontuação 1 = errou todas; pontuação 2 = acertou entre 1 e 4 lacunas; pontuação 3 = acertou entre 5 e 6 lacunas.

A partir dessas correções, o instrumento passou a ser composto por um total de 9 itens.

### Teoria da Resposta ao Item

Para a criação da medida, utilizamos um modelo da TRI, um método que permite identificar os itens que contenham maior quantidade de informação para a estimativa do LFS.

Cada participante que responde a todos os itens do teste possui um nível subjacente de habilidade de LFS. Assim, é possível atribuir uma pontuação que indica a posição do participante na escala de habilidade, representada pelo *θ* (teta) ^15^. A medida do LFS obtida pelo modelo da TRI possui escala com média zero e desvio padrão (DP) igual a 1, onde o valor estimado para cada indivíduo costuma ser interpretado como o número de DP acima ou abaixo da média. Assim, valores acima da média indicam um maior nível de LFS, enquanto valores abaixo da média indicam um menor nível de LFS. Para estimar a medida de LFS usamos o TLFS composto por 9 itens.

### Modelo de Resposta Gradual 

Usamos o Modelo de Resposta Gradual de Samejima ^16^ para criar a medida com a TRI. Esse modelo utiliza pelo menos dois parâmetros para cada item: *a*_*i*_ , que indica o poder de discriminação, e *b*_*i,k*_ que indica a dificuldade do item no caso de itens com duas categorias e a dificuldade da categoria *k* no caso de itens com mais de duas categorias. Itens cuja discriminação assumir valores maiores que 1 possuem boa capacidade de discriminação ^17^.

Para a sua aplicação adequada, é necessário que duas suposições sejam atendidas: unidimensionalidade e independência local ^18^. Se a unidimensionalidade for atendida, um único traço latente influencia as respostas dos itens, garantindo a independência local ^19^. Os modelos unidimensionais podem ser aplicados adequadamente, desde que essa suposição seja considerada de forma suficiente. A unidimensionalidade do instrumento foi avaliada por análise fatorial exploratória, utilizando uma matriz de correlação policórica, sendo considerada suficiente uma proporção de variância acumulada superior a 20% ^18^ ^,^ ^20^. 

As análises foram realizadas no software RStudio 2024.12.0, utilizando os pacotes *psych* 2.3.3 e *mirt* 1.41.

### Evidências de validade convergente

A validade convergente é demonstrada quando os escores do teste em avaliação estão altamente correlacionados com os escores de um teste que mede conceitos semelhantes ou relacionados ^21^. Neste estudo, foi feita a comparação das médias do traço latente do LFS e das classes latentes com as variáveis idade, escolaridade e classe econômica, fatores amplamente reconhecidos na literatura por influenciarem os níveis de LFS. Já foi relatado uma associação entre os baixos níveis de LFS e idade avançada ^22^, baixa escolaridade ^23^ e menor renda ^24^. A análise ocorreu por meio de uma análise de variância com teste *post-hoc* de Bonferroni e teste qui-quadrado de Pearson. Foram utilizados o software SPSS (https://www.ibm.com/) e o pacote *survey* 4.4-2 no RStudio 2024.12.0.

Todas as análises foram realizadas considerando a amostragem complexa e aplicando os pesos amostrais.

### Tamanho da amostra

O tamanho da amostra recomendado para o modelo da TRI varia conforme o modelo escolhido ^25^. Estudos com amostras de 500 participantes podem gerar estimativas confiáveis dos parâmetros dos itens e das habilidades dos sujeitos, semelhantes às obtidas em amostras maiores ^26^ ^,^ ^27^. Considerando a importância de amostras maiores para o desenvolvimento e aprimoramento das medidas utilizando a TRI ^25^, foi usada uma amostra de 1.181 participantes, garantindo a confiabilidade da análise.

### Análise de classes latentes

Para a classificação do escore gerado pela TRI, foi utilizado o modelo de classes latentes. Essa abordagem permite identificar como a associação entre um conjunto de variáveis pode ser explicada por uma variável categórica latente não observada ^28^, ou seja, permite agrupar os indivíduos em classes para as quais seus perfis de resposta aos itens tenham maior probabilidade de pertencer (Material Suplementar − Apêndice S1; https://cadernos.ensp.fiocruz.br/static//arquivo/suppl-e00060225_9313.pdf). Dessa forma, a LCA mostra como as probabilidades de um conjunto de variáveis categóricas observadas diferem entre grupos de indivíduos, sendo que a associação dos indivíduos a esses grupos não é diretamente observável ^29^. 

O resultado do agrupamento dos indivíduos foi confrontado com o nível de LFS estimado, com o objetivo de identificar pontos de corte que classifiquem os indivíduos conforme seu nível de LFS. Nesse caso, os itens do TLFS são as variáveis categóricas observadas e as classes latentes indicam diferentes categorias de LFS.

Para determinar o número de classes, foram ajustados modelos com uma, duas, três e quatro classes. Os ajustes dos modelos foram comparados utilizando o critério de informação Bayesiano (BIC, acrônimo em inglês) ^30^, entropia relativizada e a relevância prática das classificações. A entropia mede a incerteza da classificação e valores mais altos indicam melhor desempenho do modelo. Os pontos de corte foram definidos com base no índice de Youden ^31^. As análises foram realizadas no RStudio 2024.12.0, utilizando o pacote *polca* 1.6.0.1.

### Aspectos éticos

O estudo foi aprovado pelo Comitê de Ética em Pesquisa da Universidade Federal do Rio Grande do Sul (UFRGS, CAAE: 52872721.0.0000.5347) e pelos Comitês de Ética da Universidade Federal de Santa Maria (UFSM, CAAE: 52872721.0.3001.5346), Universidade Federal de Pelotas (UFPEL, CAAE: 52872721.0.3002.5317) e Universidade Estadual de Mato Grosso do Sul (UEMS, CAAE: 52872721.0.3003.8030). A pesquisa foi conduzida em conformidade com as *Resoluções n° 466/2012* e *n° 510/2016* do Conselho Nacional de Saúde. Todos os participantes assinaram o termo de consentimento livre e esclarecido (TCLE) em duas vias.

## Resultados

A amostra foi composta por 1.181 participantes, com metade dos participantes apresentando idade entre 30 e 59 anos. A maioria tinha mais de 12 anos de escolaridade e pertencia aos estratos socioeconômicos A e B (Tabela 1).

**Tabela 1:** Características sociodemográficas da amostra (n = 1.181).

Características	%
Sexo	
Feminino	50,77
Masculino	49,22
Idade (anos)	
18-29	23,50
30-59	52,21
≥ 60	24,28
Escolaridade (anos)	
0-8	26,55
9-11	13,97
≥ 12	59,48
Classificação econômica	
A/B	60,08
C	32,18
D/E	7,72

Nota: percentual ajustado por pesos amostrais.

### Teste de Letramento Funcional em Saúde

A análise de conteúdo do instrumento TLS permitiu avaliar cada questão individualmente, identificando aquelas que abordavam temas semelhantes ou conteúdos sobrepostos. Inicialmente, os pré-testes foram realizados utilizando o TLS no aplicativo REDCap. Nessa primeira etapa, foram fornecidos os cartões impressos ao entrevistado, e perguntas foram feitas oralmente pelo entrevistador. A parte textual era disponibilizada e preenchida pelo entrevistado no aplicativo. A partir do *feedback* recebido, foram identificados problemas como a dificuldade de leitura devido ao formato do texto no aplicativo, frases de difícil interpretação e tempo de aplicação muito longo. Dessa forma, o TLFS foi desenvolvido com a proposta de usar os cartões e o texto impressos. Nessa abordagem, o entrevistador fornece o material ao entrevistado e registra as respostas no aplicativo, de modo que o entrevistado não precisa utilizar o aplicativo diretamente. Esse processo permitiu ajustar as questões antes da aplicação final, assegurando que os participantes interpretassem corretamente as instruções e que o teste fosse adequado ao público-alvo.

O primeiro fator resultante da análise fatorial exploratória explicou 59,37% da variância total acumulada. Dessa forma, podemos inferir que a suposição de unidimensionalidade suficiente foi atendida. 

### Teoria da Resposta ao Item

Todos os 9 itens do TLFS apresentaram boa capacidade de discriminação, sendo que o item T3 apresentou o maior valor e o item Q5 apresentou o menor valor de discriminação (Tabela 2).

**Tabela 2:** Estimativa dos parâmetros dos itens do Modelo de Resposta Gradual.

Item	Descrição das questões	a_i_ (EP)	b_i1_ (EP)	b_i2_ (EP)
Q1	Se você toma seu primeiro comprimido às 6:00 da manhã, quando você deveria tomar o próximo?	2,686 (0,423)	-1,254 (0,104)	-
Q2	E o próximo depois desse?	2,423 (0,363)	-1,199 (0,105)	-
Q3	Quantas dessas cápsulas você deveria tomar para completar o tratamento?	1,547 (0,211)	-0,908 (0,112)	-
Q4	Se você almoça ao meio-dia e quer tomar esse remédio antes do almoço, a que horas você deve tomá-lo?	1,518 (0,224)	-1,485 (0,160)	-
Q5	Você esqueceu de tomá-lo antes do almoço, a que horas você deve tomá-lo?	1,401 (0,194)	-0,972 (0,123)	-
Q6	Quanto do antitérmico Tylenol deverá ser administrado para uma criança de 3 anos de idade com 24kg?	1,445 (0,199)	-0,970 (0,121)	-
T1	Texto: preparação para raio-x	2,217 (0,315)	-3,339 (0,395)	-1,299 (0,113)
T2	Texto: o dia anterior ao raio-x	1,911 (0,252)	-3,862 (0,524)	-0,796 (0,093)
T3	Texto: o dia do raio-x	2,725 (0,413)	-2,801 (0,269)	-1,330 (0,105)

a_i_: parâmetro de discriminação do item; b_i1_, b_i2:_ parâmetros de dificuldade de categoria do item; EP: erro padrão da estimativa.

**Figura 1:**
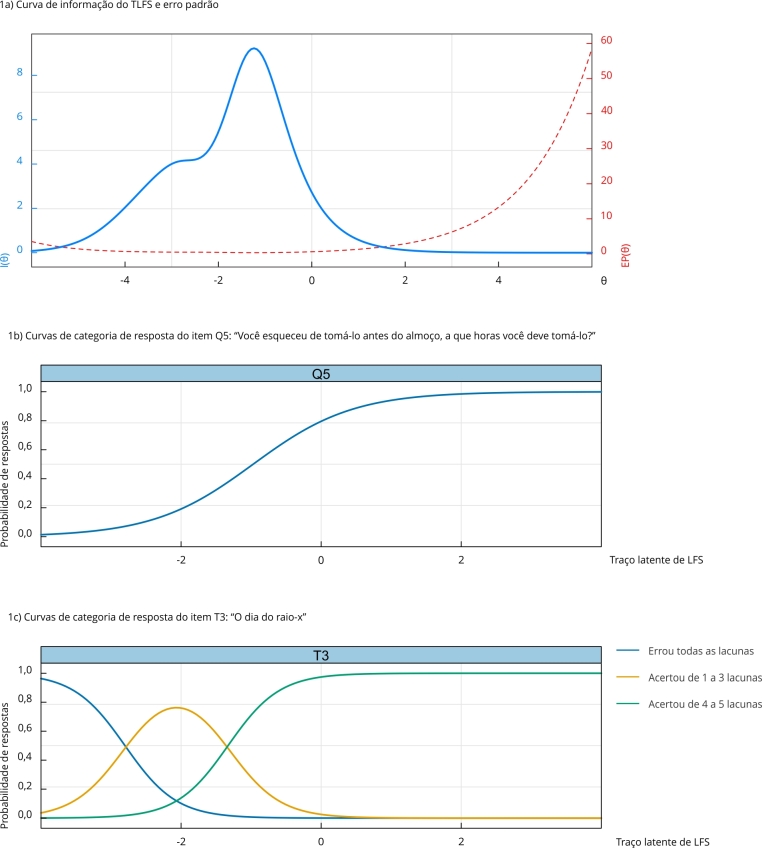
Curvas de informação e de categoria de resposta dos itens do Teste de Letramento Funcional em Saúde (TLFS).

O escore TRI gerado a partir do TLFS é mais preciso para medir indivíduos com níveis do traço latente ao redor de DP = 1,5 abaixo da média, ou seja, o escore gerado demonstra maior eficiência ao estimar o nível de letramento em saúde para participantes com níveis mais baixos de LFS (Figura 1a).

LFS: letramento funcional em saúde.

A curva característica do item ilustra a relação entre o nível do traço latente de LFS do participante e a probabilidade de concordância com a resposta ou categoria específica. A Figura 1b apresenta a probabilidade de um indivíduo acertar a questão, enquanto a Figura 1c ilustra a probabilidade de acerto em função das categorias de resposta, ambas baseadas no seu verdadeiro LFS.

### Evidências de validade convergente

A análise de evidências de validade convergente constatou uma diminuição significativa nas médias do traço latente do LFS entre os participantes com mais de 60 anos de idade, baixa escolaridade e classificados nos estratos socioeconômicos mais baixos (D e E) (Tabela 3). 

**Tabela 3:** Médias do traço latente e classificação dos participantes em três categorias de letramento funcional em saúde (LFS) em relação às variáveis idade, escolaridade e classificação econômica.

Variável	Média (DP) *	Inadequado LFS (%)	Limitado LFS (%)	Adequado LFS (%)	Valor de p **
Idade (anos)					p < 0,0001
18-29	0,108 (0,818)^a^	14,39	11,65	73,96	
30-59	0,116 (0,752)^a^	14,74	9,61	75,64	
≥ 60	-0,355 (0,891)^b^	35,69	14.42	49,89	
Escolaridade (anos)					p < 0,0001
0-8	-0,694 (0,845)^a^	51,44	15,04	33,52	
9-11	0,059 (0,702)^b^	13,44	14,18	72,39	
≥ 12	0,296 (0,644)^c^	7,08	8,89	84,03	
Classificação econômica					p < 0,0001
A/B	0,228 (0,714)^a^	10,40	8,77	80,83	
C	-0,245 (0,841)^b^	28,48	14,94	56,57	
D/E	-0,755 (0,859)^c^	56,07	15,26	28,67	

DP: desvio padrão;

* Médias seguidas de mesma letra entre as categorias de uma mesma variável não diferem, considerando 5% de significância pelo ajuste de Bonferroni;

** Teste qui-quadrado;

Nota: percentual ajustado por pesos amostrais.

### Análise de classes latentes

O modelo de três classes latentes apresentou o melhor ajuste (BIC: 10193.52; entropia relativizada: 0.9967). A entropia relativizada aumentou com o aumento no número de classes (Material Suplementar − Tabela S1; https://cadernos.ensp.fiocruz.br/static//arquivo/suppl-e00060225_9313.pdf). Entretanto, o modelo de três classes parece ser o mais adequado, considerando a pouca diferença em relação ao modelo de quatro classes. Além disso, a divisão em três categorias é a mais comumente utilizada em instrumentos de LFS. 

A partir da classificação em três classes latentes, foram definidos três níveis de LFS. O índice de Youden foi usado para estabelecer os pontos de corte no escore gerado pela TRI, categorizando em inadequado LFS (até -0,7507), limitado LFS (entre -0,7507 e -0,393) e adequado LFS (acima de -0,393).

A análise de comparação das classes indicou que participantes com maior idade, menor escolaridade e renda mais baixa tendem a apresentar LFS inadequado. Em contrapartida, os participantes mais jovens (menos de 60 anos), com maior escolaridade (mais de 12 anos de estudo) e maior renda, em sua maioria, foram classificados com LFS adequado (Tabela 3).

### Aplicativo para uso do TLFS

Para facilitar a aplicação do instrumento e considerando a inviabilidade de realizar os cálculos do escore TRI manualmente para gerar o traço latente, o TLFS foi disponibilizado na plataforma Shiny (disponível em: https://letramentoemsaude.shinyapps.io/inicio/). A ferramenta pode ser acessada e utilizada tanto para pesquisa quanto para aplicação na prática clínica.

## Discussão

Neste estudo, utilizamos a análise da TRI para desenvolver a medida de um traço latente que mede o LFS, a partir da redução de um instrumento amplamente utilizado. Como resultado, foi criado o instrumento TLFS, composto por questões que avaliam o conhecimento em leitura e numeramento no contexto da saúde. As 22 questões que compõem o instrumento foram agrupadas de acordo com os critérios de independência local da TRI, totalizando 9 itens, que apresentaram boas evidências de validade conforme as análises de validade convergente.

A partir da análise da TRI, foi possível estimar os parâmetros de discriminação e dificuldade dos itens. De maneira geral, os itens apresentaram parâmetros de discriminação classificados como altos ou muito altos, conforme os critérios de Baker ^15^. Esses resultados demonstram que todos os itens do instrumento apresentam uma contribuição relevante na estimativa do traço latente, não havendo necessidade de exclusão de nenhum item.

Os itens Q1, Q2 e T3 apresentaram os maiores valores de discriminação, indicando que são os itens mais informativos para distinguir indivíduos com diferentes níveis de habilidade de LFS. Destacam-se as questões Q1 “Se você toma seu primeiro comprimido às 6:00 da manhã, quando você deveria tomar o próximo?” e Q2 “E o próximo depois desse?”, que demandam habilidades de adição básica. 

A curva de informação do TLFS demonstrou maior precisão para medir indivíduos com níveis do traço latente ao redor de 1,5 DP abaixo da média, confirmando que o teste favorece a identificação de indivíduos com nível mais baixo de LFS. Considerando essa característica, o TLFS pode constituir um instrumento promissor para a realização de triagens de populações com LFS inadequado. Diversos estudos já demonstraram a efetividade de intervenções voltadas ao letramento em saúde em diferentes populações ^32^ ^,^ ^33^, trazendo benefícios relacionados à promoção da saúde, prevenção de doenças e cuidado em saúde ^34^. Dessa forma, a identificação de indivíduos com níveis mais baixos de LFS é fundamental para o planejamento de intervenções direcionadas à melhoria do letramento em saúde.

As curvas de categoria de resposta do item T3 demonstraram um número adequado de categorias de resposta, no qual todos os itens apresentaram um pico bem definido e as curvas não ficaram sobrepostas. Foi observado que indivíduos com baixos níveis de LFS têm maior probabilidade de errar todas as lacunas ou acertar de 1 a 3 colunas, enquanto indivíduos com níveis elevados de LFS tendem a acertar de 4 a 5 lacunas. Esse padrão de resposta é esperado, pois indica que as opções de resposta representam adequadamente o conteúdo de cada item.

O parâmetro de dificuldade localiza o ponto na escala de habilidade onde o item funciona melhor ^15^. Dessa forma, embora os itens apresentem uma variação de -0,796 a -3,862, todos os itens identificaram melhor indivíduos com baixas habilidades em LFS.

Além disso, a variação observada nos parâmetros de dificuldade reforça a importância de incluir itens que abordem aspectos distintos do LFS, permitindo a mensuração de diferentes níveis de dificuldade do TLFS. Entre os itens com maior parâmetro de dificuldade, destacam-se os itens T2, Q6 e Q5. O item Q6 “Quanto do antitérmico Tylenol deverá ser administrado para uma criança de 3 anos de idade com 24kg?” requer a leitura e interpretação de intervalos em uma tabela. Já o item Q5 “Você esqueceu de tomá-lo antes do almoço, a que horas você deve tomá-lo?” envolve a interpretação de instruções de uso de medicamentos, associada ao cálculo de adição de horas. Esses parâmetros sugerem que habilidades comumente necessárias para interpretar e seguir uma prescrição medicamentosa representam uma tarefa desafiadora para uma proporção relevante da população estudada.

Como diversos instrumentos são utilizados para avaliar o LFS, a coleta de evidências de validade convergente foi examinada por meio da comparação da medida gerada pelo TLFS com fatores associados previamente estabelecidos na literatura. Estudos anteriores, realizados em outros países, indicam uma associação entre os níveis de LFS e fatores como idade ^22^, escolaridade ^23^ e renda ^24^. No Brasil, essa associação já foi demonstrada em pesquisas anteriores. Estudos apontam que níveis mais baixos de LFS estão relacionados à idade mais avançada ^35^ ^,^ ^36^ ^,^ ^37^, baixa escolaridade ^35^ ^,^ ^36^ ^,^ ^37^ ^,^ ^38^ ^,^ ^39^ ^,^ ^40^ e menor renda ^35^ ^,^ ^37^. Os achados do nosso estudo demonstraram as primeiras evidências de validade convergente ao reforçar essa relação nos três fatores avaliados, fornecendo informações que confirmam a precisão das medidas do TLFS.

A análise de classes latentes foi utilizada para criar as categorias de TLFS e o modelo de três classes foi escolhido com base nas medidas de ajuste e aplicabilidade prática das classificações. Dessa forma, foram identificadas três categorias, nomeadas como inadequado, limitado e adequado LFS. As análises de evidências de validade convergente confirmaram essa classificação, demonstrando consistência com os padrões observados nas médias dos escores.

Algumas limitações do estudo devem ser consideradas na avaliação do instrumento. Embora a amostra tenha sido de tamanho adequado para a aplicação da TRI, ela se restringiu a residentes de cidades das regiões Sul e Centro-oeste do Brasil, limitando sua representatividade nacional. Além disso, a amostra foi composta predominantemente por indivíduos com níveis elevados de escolaridade e renda, o que pode ter influenciado nos resultados, uma vez que os itens apresentaram níveis de dificuldade abaixo da média para a maioria dos participantes. Nesse sentido, a medida desenvolvida pode ser mais adequada para esse perfil.

A partir desse estudo, foram obtidas as primeiras evidências de validade do TLFS. O instrumento demonstrou bom desempenho psicométrico para avaliar o nível de LFS de um indivíduo, apresentando evidências de validade convergente, além de boas estimativas de discriminação dos itens e uma curva de informação precisa. O tempo de aplicação do instrumento foi reduzido, pelo menos, à metade em comparação com o TLS, ainda que não tenhamos cronometrado para toda a amostra. A inserção do TLFS no aplicativo Shiny permite avaliar o nível de LFS de forma ágil, possibilitando que o profissional da saúde ou pesquisador identifique o perfil de LFS do indivíduo e forneça orientações personalizadas, conforme as necessidades específicas de cada pessoa. Essa ferramenta facilita a aplicação do instrumento tanto na prática clínica quanto em pesquisas, auxiliando no acompanhamento e intervenções de forma individualizada.

## Conclusão

O TLFS oferece uma medida confiável e viável para aplicação em serviços de saúde e pesquisa que exigem agilidade. Devido à sua praticidade, o TLFS pode ser útil na triagem do LFS de usuários dos serviços de saúde, possibilitando uma avaliação rápida do nível de LFS em ambientes de saúde onde existe uma alta demanda e o tempo de atendimento é limitado. Tendo em vista que este é o primeiro estudo a aplicar a TRI na análise do TLFS, investigações futuras que aprofundem a compreensão dos padrões de resposta aos itens e avaliem outros tipos de validade contribuirão significativamente para o aprimoramento do instrumento. Além disso, o TLFS pode fornecer uma medida adequada em estudos que investigam a associação entre LFS e desfechos em saúde.

## Material Suplementar

Material Suplementar
