# Supplementary material for: Development of a measure of functional health literacy: application of Item Response Theory
Source: Cad Saude Publica. 2026 Feb 13;42:e00060225. [Article in Portuguese] doi: 10.1590/0102-311XPT060225 (PMC12928199; doi:10.1590/0102-311XPT060225)
Supplement: Material Suplementar [file 1678-4464-csp-42-PT060225-s.pdf]

Material Suplementar

Figura S1 Materiais de aplicação do Teste de Letramento Funcional em Saúde.

Material Suplementar 1

CARTÃO 1

Paciente: Angélica de Souza

TETRACICLINA 500mg----------------- 40 cáps.

Tomar uma cápsula de 6/6h.

Importante: Utilize todo este medicamento.

Dr. Eduardo Silveira
CRM: 956827

CARTÃO 2

Paciente: Angélica de Souza

DOXICICLINA 100mg----------------- 20 comp.

Tome o medicamento de estômago vazio 1 hora antes ou 2-3 horas após uma refeição.

Dr. Eduardo Silveira
CRM: 956827

CARTÃO 3

A dose do antitérmico Tylenol®, suspensão oral, a ser administrada em crianças varia de acordo com a tabela abaixo:

**Antitérmico Tylenol® para Criança Suspensão Oral:**	
**Peso (Kg)**	**Dose (mL)**
11-15	5
16-21	7,5
22-26	10
27-31	12,5
32-43	15
Para crianças abaixo de 11Kg ou 2 anos, consulte seu médico.	

Na próxima parte do teste você encontrará um texto abordando orientações para exame de raio-x. As frases estão incompletas, mas juntas formam um texto. Colocamos um espaço em branco com uma linha (________) no lugar da palavra que está faltando. Apresentamos, abaixo, 4 alternativas de resposta. Escolha uma alternativa e diga em voz alta para o entrevistador marcar a alternativa escolhida no aplicativo.

PREPARAÇÃO PARA RAIO X

O seu médico mandou você fazer um raio X ________________.

- ☐ do estômago
- ☐ do diabetes
- ☐ dos pontos
- ☐ dos germes

Você deve estar com o estômago________________quando vier para ________________.

- ☐ asmático
- ☐ vazio
- ☐ contente
- ☐ anêmico

- ☐ o mercado
- ☐ o almoço
- ☐ o dormitório
- ☐ o exame

O raio X________________de 1 a 3________________para ser realizado.

- ☐ levará
- ☐ verá
- ☐ falará
- ☐ olhará

- ☐ camas
- ☐ cérebros
- ☐ horas
- ☐ dietas

O DIA ANTERIOR AO RAIO X

No jantar, coma apenas um____________lanche, como uma____________, acompanhada de café ou chá.

- ☐ pequeno
- ☐ suco
- ☐ ataque
- ☐ nauseante

- ☐ dedos
- ☐ garganta
- ☐ torrada
- ☐ coxa

Após____________da véspera, você não deve ____________ ou beber nada,

- ☐ um minuto
- ☐ a meia-noite
- ☐ durante
- ☐ antes

- ☐ fácil
- ☐ comeu
- ☐ bebeu
- ☐ comer

_____________em jejum até a hora do_____________.

- ☐ comendo
- ☐ absolutamente
- ☐ cada
- ☐ permanecendo

- ☐ exame
- ☐ horário
- ☐ dia
- ☐ estado

O DIA DO RAIO X

Não tome __________ .

- ☐ consulta
- ☐ consulta sem hora marcada
- ☐ café da manhã
- ☐ clínica

Não_________, nem mesmo_____________.

- ☐ dirija
- ☐ beba
- ☐ vista
- ☐ dose

- ☐ coração
- ☐ respiração
- ☐ água
- ☐ câncer

Se você tiver qualquer_____________, ligue para______________de raio X no 3616-4500.

- ☐ resposta
- ☐ exercício
- ☐ aparelho
- ☐ pergunta

- ☐ o Departamento
- ☐ a Distensão
- ☐ a Farmácia
- ☐ a Dor de dente

Apêndice S1 Definição do ponto de corte no nível de letramento funcional em saúde

Ajuste do modelo de classes latentes para três classes [1].

$$P(Y=y)=\sum_{c=1}^{3}\gamma_c\prod_{m=1}^{9}\prod_{k=1}^{3}\rho_{\text{mk}\vee c}^{I(y_m=k)}$$

onde,

Y → possíveis perfis de resposta aos 9 itens do instrumento de medida Teste de Letramento Funcional em Saúde (como são seis itens com duas categorias de resposta e três itens com três categorias de resposta, existem 1.728 perfis possíveis);

$P(Y=y)$ → probabilidade de um indivíduo apresentar especificamente um perfil de respostas y ;

$I(y_m=k)$ → função indicadora que será 1 se o indivíduo respondeu a categoria *k* do item *m*;

γ_c → proporção populacional (ou prevalência) de indivíduos na categoria *c* da variável latente Classe de letramento funcional em saúde, com $c=1$ (indivíduos com menor letramento funcional em saúde), $c=2$ (indivíduos com letramento funcional em saúde intermediário) ou $c=3$ (indivíduos com maior letramento funcional em saúde);

$\rho_{\text{mk}\vee c}$ → probabilidade de responder a categoria *k* ao *m-ésimo* item condicionado à classe *c*, isto é, de acordo com a categoria de letramento funcional em saúde na qual o indivíduo se encontra, indica qual a probabilidade de responder a categoria *k* do *m-ésimo* item;

Após o ajuste do modelo de classes latentes, é possível estimar a probabilidade a posteriori (p_{ic}), ou seja, a probabilidade do indivíduo *i* pertencer a classe *c* de acordo com seu perfil de resposta aos 9 itens do instrumento de medida Teste de Letramento Funcional em Saúde:

$$p_{\text{ic}}=\frac{\left(\prod_{m=1}^{9}\prod_{k=1}^{3}\rho_{\text{mk}\vee c}^{I(y_m=k)}\right)\gamma_c}{\sum_{c=1}^{3}\gamma_c\prod_{m=1}^{9}\prod_{k=1}^{3}\rho_{\text{mk}\vee c}^{I(y_m=k)}}$$

Finalmente, o indivíduo será alocado para a classe latente na qual ele obtiver maior probabilidade de pertencer. Quanto maior for esta probabilidade, menor será a incerteza de classificação.

Referência

1. Borges RB. Análise de classes latentes: uma aplicação visando definir pontos de corte na medida gerada na escala Brief Measure of Emotional Preoperative Stress [Dissertação de Mestrado]. Porto Alegre: Universidade Federal do Rio Grande do Sul; 2019.

Tabela S1 Medidas de ajuste para modelos de classes latentes com diferentes quantidades de classes.

Número de classes	BIC	Entropia bruta	Entropia relativizada
**1**	11.634,14	4,889607	-
**2**	10.264,8	4,296656	0,9947513
**3**	10.193,52	4,217849	0,9967492
**4**	10.193,77	4,173238	0,997451
